# Self-stigma profiles in schizophrenia: a Latent Class Analysis approach ^*^

**DOI:** 10.1590/1518-8345.7504.4593

**Published:** 2025-07-11

**Authors:** Alejandra Caqueo-Urízar, Felipe Ponce-Correa, Alfonso Urzúa, Guillaume Fond, Laurent Boyer

**Affiliations:** 1 Universidad de Tarapacá, Instituto de Alta Investigación, Arica, Arica y Parinacota, Chile.; 2 Universidad de Tarapacá, Escuela de Psicología y Filosofía, Arica, Arica y Parinacota, Chile.; 3 Universidad Católica del Norte, Escuela de Psicología, Antofagasta, Antofagasta, Chile.; 4 Aix-Marseille Université, Public Health, Chronic Diseases and Quality of Life - Research Unit, Marseille, Provence-Alpes-Côte d’Azur, France.

**Keywords:** Schizophrenia, Social Stigma, Latent Class Analysis, Mental Health Services, Sociodemographic Factors, Psychiatric Status Rating Scales

## Abstract

this study aimed at analyzing the internalized stigma latent profiles and the covariates that predict variations in their levels considering antecedent variables such as ethnicity, gender and some relevant clinical characteristics like premorbid adjustment, Duration of Untreated Psychosis and symptoms.

Latent Class Analysis (LCA) was used to devise a solution with three internalized stigma profiles in a sample comprised by 227 patients diagnosed with schizophrenia from the Public Mental Health Centers of the city of Arica, Chile.

the results showed that premorbid adjustment is a significant predictor of class belonging for the latent stigma profiles. When analyzing the sociodemographic characteristics and contrary to what was hypothesized, ethnicity was not a relevant predictor of internalized stigma profiles.

the latent classification model is suitable for assessing stigma profiles in order to target future interventions in specific foci and at-risk populations.

## Highlights

**(1)** Latent Class Analysis revealed different internalized stigma profiles in schizophrenia: high, moderate and low. **(2)** Ethnic identity was not a significant predictor: contrary to common assumptions, belonging to any given ethnic group did not exacerbate internalized stigma in individuals with schizophrenia. **(3)** Premorbid adjustment was a key factor: poor psychosocial functioning before psychosis onset was a significant predictor of high and moderate stigma profiles. **(4)** Severity of the symptoms was irrelevant: severity of the psychotic symptoms was not correlated with internalized stigma, suggesting that the psychosocial factors are more influential. **(5)** Targeted interventions are required: individuals with poor premorbid adjustment may benefit from stigma-reduction programs focused on improving their social and coping skills.

## Introduction

The study of internalized stigma in population groups with severe mental disorders has gained special relevance in the last decades^(1-2)^. People exposed to environments and communities where the stigma of a mental disorder is present end up internalizing negative preconceptions and stereotypes that affect their identity and exert negative impacts on their mental health and on multiple clinical outcomes^(3-5)^, including increased depressive symptoms^(6)^, suicidal ideation^(7-8)^, impaired self-esteem^(9-10)^, decreased quality of life^(11)^, weakened social relationships^(12)^, and difficulties in empowerment^(13)^, resilience^(14)^, treatment adherence^(15)^ and access to psychiatric care^(16)^. A relationship between stigma and medication side effects was also found^(17)^.

In severe mental disorders, the increase in self-stigma reduces the capacity for recovery^(11)^, hindering remission of the symptoms^(18)^, decreasing functionality and the capability to maintain social roles^(19)^ and exerting negative impacts on a person’s subjectivity, thus losing hope and self-esteem^(9-10)^. Other factors such as autism spectrum symptoms in the schizophrenic population and gender also play a role, with women tending to experience higher stigma levels^(20)^.

Although the consequences of self-stigma have been well explored, its risk factors and social determinants have not been studied in depth. Previous studies have shown that the discrimination experienced by people with schizophrenia belonging to ethnic minorities or from low socioeconomic backgrounds increases the risk of internalized stigmatization^(21)^. On the other hand, patients with psychosis that have been untreated for longer periods of time are at a higher risk of self-stigma^(22)^. It has also been established that stigma can raise the threshold for treatment initiation, especially when it comes from family members^(23)^; therefore, the direction of this relationship and the mechanisms that constitute it still require further development. Premorbid poor social functioning has also been found to increase the risk of internalized stigmatization in schizophrenia^(14)^. Premorbid maladaptation severely limits socio-cognitive development, both in the ability to interpret the social world and in internal psychological states^(24)^, which reduces a patient’s ability to cope with negative meanings coming from the social world.

Despite the growing body of literature on self-stigma, the interplay of multiple risk factors (such as discrimination, family dynamics and premorbid functioning) remains insufficiently researched, particularly in Latin American populations. In these regions, the scarcity of mental health professionals and psychiatrists further complicates implementing standardized tools for assessing self-stigma. This study aims at identifying the demographic, psychosocial and clinical factors that contribute to high self-stigma levels among individuals with schizophrenia in Latin America. It is hypothesized that factors such as ethnicity, gender, premorbid adjustment, Duration of Untreated Psychosis (DUP) and severity of the psychotic symptoms will significantly predict self-stigma in this population group. Specifically, ethnicity may influence stigma experiences through cultural identity, while gender might affect perception and internalization of stigma due to gender-based differences. Premorbid adjustment, which includes social relationships, peer interactions, academic performance, occupational functioning and social-sexual engagement prior to schizophrenia onset, may also play a key role in shaping self-stigma. Additionally, longer Durations of Untreated Psychosis and more severe symptoms are expected to contribute to heightened self-stigma levels. By exploring these factors within the context of local cultural and healthcare settings, this study seeks to provide valuable insights into the development and persistence of self-stigmatization in this population segment.

## Methods

### Study design

This study used a quantitative cross-sectional design with a correlational approach, aiming to investigate the associations between internalized stigma and various clinical, sociodemographic, and psychosocial factors in individuals diagnosed with schizophrenia. The cross-sectional nature of the study allowed assessing relationships between variables at a single moment in time, providing a snapshot of the factors influencing internalized stigma in this population group.

### Setting

This study was carried out in the 3 Public Mental Health Service centers from Arica, Chile. The participating centers are part of the State’s Mental Health service; consequently, the assistance provided to the patients is free of charge, and they also receive free antipsychotic drugs. The State has a health plan that covers the care of this psychiatric disorder by law. Treatment is initiated after the first psychotic outbreak, when the patient is admitted to the Psychiatric Unit of the Day Hospital and, once compensated, continues their treatment on an outpatient basis in any of these three services depending on geographical proximity. The patients are usually followed up monthly.

### Participants

The participants were individuals diagnosed with schizophrenia, as per the International Classification of Diseases-Tenth Revision (ICD-10) criteria. Non-probability sampling by availability was used. The participants had to meet the following criteria to be included in the study.

#### Inclusion criteria

The study included all stabilized community-dwelling patients diagnosed with Schizophrenia according to the criteria set forth in the International Classification of Diseases (ICD)-10th version. The treating psychiatrist provided this information.

#### Exclusion criteria

Patients with a history of neurological disorders (including stroke, epilepsy, and head injury) or any disease affecting the Central Nervous System were not included in the current study to ensure that all subjects would be able to participate in the interviews fully. All patients have medical tests in their clinical history that rule out any organic cause for the psychotic behaviors.

As most of the patients were stable in relation to their psychotic symptoms, the number of subjects excluded was low; in addition, the overwhelming majority agreed to participate.

### Variables and instruments

#### Outcome variable

The primary study outcome was internalized stigma, which was assessed using the Internalized Stigma of Mental Illness (ISMI-29) Scale^(25)^: ISMI is a 29-item self-report questionnaire designed to measure internalized stigma across five subscales: Alienation, Endorsement of Stereotypes, Perceived Discrimination, Social Withdrawal, and Resistance to Stigma. Higher total scores in ISMI indicate more severe internalized stigma. The Spanish version of ISMI was used^(26)^.

#### Predictive variables

Positive and Negative Syndrome Scale (PANSS) for Schizophrenia^(27)^: PANSS is a 30-item scale used to assess psychotic symptoms in individuals with schizophrenia, divided into five factors: Positive, Negative, Excitement, Depression, and Cognition^(28)^. PANSS was translated and validated to Spanish^(29)^.

Premorbid Adjustment Scale (PAS)^(30)^: This scale uses retrospective interviews to assess the patients’ psychosocial development prior to psychosis onset, focusing on five domains: Sociability and withdrawal, Peer relationships, Scholastic performance, Adaptation to school, and Social-sexual adjustment. Higher PAS scores indicate poorer premorbid adjustment. The scale was adapted to Spanish^(31)^.

Duration of Untreated Psychosis (DUP)^(32)^: DUP was defined as the time from the onset of the first acute psychotic episode to initiation of the antipsychotic treatment. The DUP score was calculated based on information provided by the patients, their primary caregivers and the medical records.

#### Potential confounders

Sociodemographic variables such as ethnicity and biological sex were considered as potential confounders in the analysis. Ethnicity was assessed through self-report, with the participants categorizing themselves as belonging to some ethnic group (1) or not (2). This categorization reflects cultural and identity-based distinctions relevant to stigma experiences. Biological sex was recorded as a binary variable (1 = Male; 2 = Female), based on the participants’ self-identification. These sociodemographic variables were included in the analysis to account for their potential influence on self-stigma, as both ethnicity and gender have been shown to exert impacts on stigma-related experiences in various Mental Health contexts.

In addition to the primary variables of interest, several sociodemographic and clinical characteristics were collected to enhance the study sample description. While these variables were not directly related to the hypothesis, they provided valuable context for understanding the population group under study.

Age was recorded as a continuous variable, allowing for a detailed demographic profile of the sample. Relationship status was assessed using a binary variable, where the participants indicated whether they were currently in a relationship or not (Yes = 1, No = 0). Both age at onset of the first acute psychotic episode and age at treatment initiation were recorded as continuous variables.

### Data sources/measurement

The primary data were collected through structured, face-to-face interviews conducted by duly trained psychologists. Each participant was assessed individually in a private setting at their respective Mental Health center. The ISMI-29, PAS and PANSS scales were applied as part of the interview process, with additional information on DUP gathered from medical records, patients’ self-reports and caregivers’ inputs. All measuring instruments used in the study are well-established and have already been validated for use in Spanish-speaking populations. These tools have shown good reliability and validity in previous research studies.

### Bias

Several measures were taken throughout the study to minimize potential bias sources. Selection bias was mitigated by including participants from three public Mental Health centers, which provided a broad range of clinical experiences. However, the non-probability sampling method (convenience sampling) limits generalizability of the findings to the broader population of individuals with schizophrenia. Measurement bias was minimized by standardizing the interview procedures, with all assessments conducted by two trained psychologists under supervision by the main researcher. In order to address recall bias, structured interview protocols were used to guide the participants’ recall, and medical records were consulted for corroborative information when available.

### Study size

A total of 227 patients diagnosed with schizophrenia were recruited for the study. The sample size was determined based on the feasibility of patient recruitment at the three centers and the number of eligible participants attending regular follow-up appointments. This sample size was deemed adequate to achieve sufficient statistical power for the planned analyses, including Latent Class Analysis (LCA) and multinomial logistic regression, to detect meaningful associations between the predictive variables and internalized stigma.

Over a three-month period, the patients attending their monthly follow-up appointments at the primary public outpatient treatment centers for schizophrenia were invited to participate. Two psychologists, who were part of the research team and were under the supervision of the main researcher, conducted the assessments in the patients’ respective Mental Health centers. The evaluation sessions lasted between 40 and 60 minutes.

Before initiating data collection, all participants provided their informed consent in writing. The study objectives were explained to all participants, as well as the voluntary nature of their participation.

### Statistical methods

The data were analyzed using Latent Class Analysis (LCA)^(33)^, a statistical method designed to identify different subgroups or profiles within a population segment based on the answer patterns found in ISMI-29. The number of latent classes was determined using a combination of model fit indices, including the Bayesian Information Criterion (BIC), the Akaike Information Criterion (AIC) and the adjusted Bayesian Information Criterion (aBIC)^(33-34)^. Lower values in these indices indicate better-fitting models. Additionally, the Lo-Mendell-Rubin adjusted likelihood ratio test and the bootstrap likelihood ratio test were employed to assess fit of the models.

Multinomial logistic regression was used to examine the association between internalized stigma profiles and several predictive variables such as ethnicity, gender, premorbid adjustment, DUP and severity of the psychotic symptoms. This approach allowed analyzing multiple predictors and potential confounders simultaneously. In addition, subgroup analyses were performed to explore whether the relationships between predictors and stigma profiles differed by sociodemographic characteristics^(34)^.

The missing data were addressed using multiple imputation, and sensitivity analyses were conducted to assess robustness of the results under different assumptions regarding the missing data. All analyses were performed in the Mplus software (Version 8.5)^(35-36)^.

### Ethical considerations

The study was approved by the University of Tarapacá Ethics Committee (18/2009) and by the Chilean National Health Service. Written informed consents were obtained from both the patients and their primary caregivers. The study emphasized the voluntary nature of their participation and no financial compensation was provided to the participants.

## Results

The results corresponding to the descriptive analysis of the sample showed that the mean age was 41.1 years old [Standard Deviation (SD)=16.34], with 129 (56.8%) male patients, 181 (79.7%) having no partner and 86 (37.9%) self-reporting as belonging to the Aymara ethnic group. The participants’ age at the onset of the first acute psychotic episode and at treatment initiation was 21.4 years old (SD=8.4) and 25 years old (SD=8.9), respectively.

**Figure 1 -:**
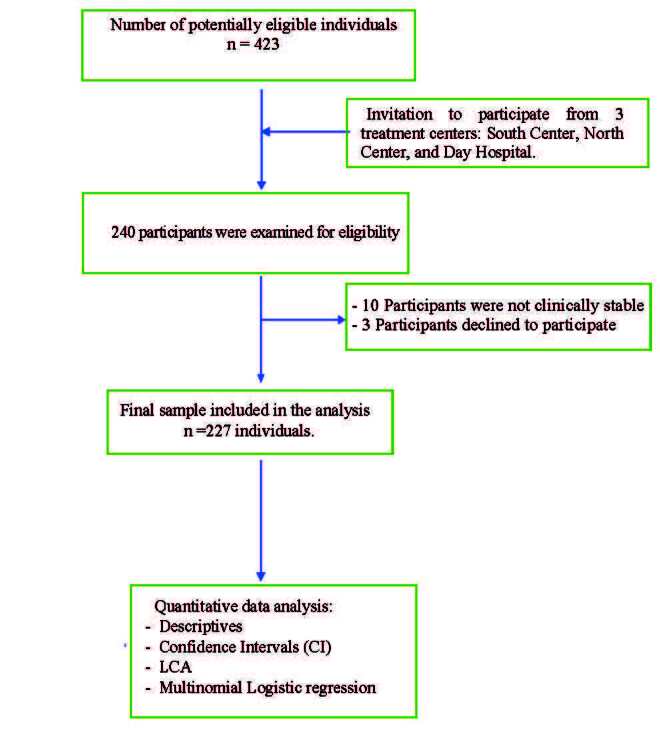
Flowchart showing the sample selection and inclusion process

The flowchart (see Figure 1) provides a clear visual representation of the process to select the participants, starting with a total number of 423 potential candidates among all three treatment centers. After screening for eligibility, 240 individuals were assessed for inclusion. Ten subjects were excluded due to clinically significant symptoms that prevented their appropriate participation in the study. Three individuals refused to take part after being invited. Thus, a final sample of 227 individuals with schizophrenia that met the inclusion criteria and provided their informed consent was included for analysis. The flowchart illustrates this process step-by-step, ensuring transparency in recruitment and inclusion of the participants.

The LCA results supported the three-class model as having the best relative fit indicators [AIC=1,976.884; aBIC=1,982.509; aLMR=89.914; Bayesian Linear Regression **(BLR)=92.676]. Entropy for the three-class model showed an adequate level** (0.82).

Table 1 contains the internalized stigma mean scores in each of the ISMI-29 dimensions and the distribution of the covariates. The patients with high stigma latent profiles were 50% male, and 47.9% identified themselves as belonging to some ethnic group. They also presented the worst mean premorbid adjustment (13.75), the longest DUP in years (3.85) and the worst symptoms in the sample (63.45). On the other hand, the moderate stigma latent profile was mostly comprised by men (62.4%) and by people who identified themselves as belonging to some ethnic group (58.4%). It also presented intermediate levels of premorbid adjustment (10.82), untreated psychosis in years (1.88) and symptoms (61.83). Finally, the low stigma latent profile consisted mostly of men (61.5%) and of people who identified themselves as belonging to some ethnic group (59.6%). It presented the best levels of premorbid adjustment (9.45), untreated psychosis in years (1.58) and symptoms (59.54).

**Table 1 -:** Descriptive statistics corresponding to the clinical variables. Arica, Arica y Parinacota, Chile, 2023

Mean (SD*)					
**ISMI** ^†^		**Total sample**	**High stigma**	**Moderate stigma**	**Low stigma**
Alienation		13.34 (4.22)	2.69 (0.06)	2.218 (0.06)	1.359 (0.06)
Perceived discrimination		11.26 (3.68)	2.87 (0.05)	2.131 (0.07)	1.363 (0.07)
Self-stigma		14.60 (4.71)	2.66 (0.04)	1.974 (0.07)	1.243 (0.06)
Resistance to stigma		12.80 (2.92)	2.33 (0.06)	2.659 (0.06)	2.867 (0.10)
Social withdrawal		13.45 (5.06)	2.84 (0.07)	2.172 (0.11)	1.270 (0.07)
(SD*)/Frequency (%)					
**Covariates**		**Total sample (n=227)**	**High stigma (n=98)**	**Moderate stigma (n=77)**	**Low stigma (n=52)**
Gender	Male	129 (56.8%)	49 (50%)	48 (62.4%)	32 (61.5%)
	Female	98 (43.2%)	49 (50%)	29 (37.6%)	20 (38.5%)
Ethnic group	Yes	123 (54.2%)	47 (47.9%)	45 (58.4%)	31 (59.6%)
	No	104 (45.8%)	51 (52.1%)	32 (41.6%)	21 (40.4%)
Premorbid adjustment		11.52 (4.45)	13.75 (4.30)	10.82 (4.01)	9.45 (3.70)
Duration of Untreated Psychosis		2.46 (6.36)	3.85 (8.76)	1.88 (5.17)	1.58 (3.11)
Symptoms		61.80 (18.91)	63.45 (16.19)	61.83 (21.64)	59.5 (19.43)

*SD = Standard Deviation; ^†^ISMI = Internalized Stigma of Mental Illness Scale

**Figure 2 -:**
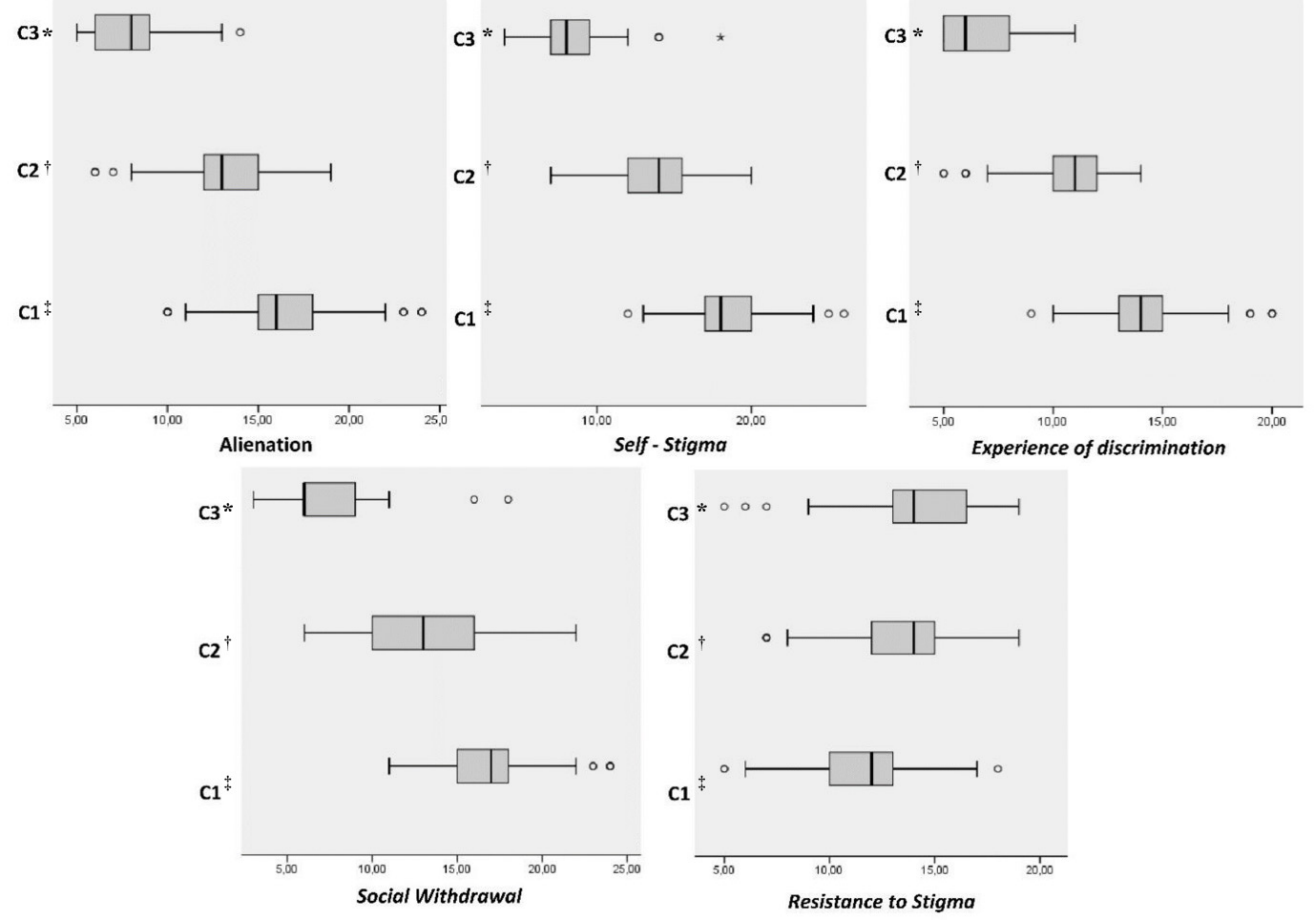
Mean values of internalized stigma latent profiles according to the ISMI-29 dimensions

Figure 2 shows the distribution of the latent profiles using a boxplot for each of the ISMI-29 classes and dimensions. It can be stated that the subjects with high self-stigmatizing tendencies and lower resistance to stigma, who have higher propensity to agreeing to negative stereotypes about schizophrenia, who have experienced more discrimination and with higher social isolation levels (n=98) were grouped in (C1). On the other hand, people with average levels in all ISMI-29 dimensions were included in (C2) (n=77). In turn, in (C3) we find participants with greater resistance to stigma and lower self-stigmatizing tendencies, capable of having a positive view about themselves and the disease, with low perceived discrimination and greater social participation (n=52).

*C3 = Low stigma; ^†^C2 = Moderate stigma; ^‡^C1 = High stigma

Table 2 presents the multinomial logistic regression results, examining the association between covariates and latent class membership for internalized stigma profiles. The low stigma profile is used as the reference group for comparison.

**Table 2 -:** Multinomial logistic regression results for the latent class covariates. Arica, Arica y Parinacota, Chile, 2023

**Reference group**	**Comparison group**	**Ethnic group**	**Gender**	**Duration of Untreated Psychosis**	**Premorbid Adjustment**	**Symptoms**
OR* (CI ^†^ )						
High stigma	Moderate stigma	1.09(0.76; 1.56)	0.34(0.04; 2.92)	0.91(0.79; 1.06)	0.87(0.71; 1.06)	0.94(0.88; 1.01)
	Low stigma	0.88(0.65; 1.19)	0.33(0.06; 1.71)	0.98(0.89; 1.08)	0.70(0.58; 0.85) ^‡^	0.98(0.93; 1.04)
Low stigma	Moderate stigma	1.23(0.86; 1.76)	1.04(0.22; 4.8)	0.93(0.80; 1.08)	1.23(1.00; 1.5) ^‡^	0.95(0.90; 1.01)

Note: The low stigma profile was used as a reference group for comparison; *OR = Odds Ratio; ^†^CI = Confidence Interval; ^‡^Statistically significant

As shown in Table 2, premorbid adjustment was the only significant predictor of membership in both the high stigma and the moderate stigma profiles. Specifically, poorer premorbid adjustment (OR=0.70 for high stigma and OR=1.23 for moderate stigma) increases the likelihood of a person being classified in either the high or moderate internalized stigma profiles, when compared to the low stigma group.

This suggests that individuals with worse psychosocial adjustment prior to psychosis onset are more likely to experience higher internalized stigma levels, as compared to those with better premorbid adjustment, who are more likely to fall into the low stigma category.

## Discussion

The Latent Class Analysis (LCA) performed in this study identified three different internalized stigma profiles in individuals diagnosed with schizophrenia: high, moderate, and low. This three-profile classification proved to be an effective model for categorizing the participants based on their latent stigma scores. Contrary to our initial hypothesis, ethnicity did not emerge as a significant predictor of belonging to the high-stigma profile. This finding challenges some prevailing assumptions in the literature on stigma. While previous research has oftentimes emphasized the relationship between belonging to some ethnic group and stigma exacerbation^(1)^ and contrary to what was hypothesized, having schizophrenia and belonging to a vulnerable social segment such as some ethnic group^(13)^ do not probably increase stigma, rejecting the combination of conditions hypothesized. The current results suggest that, in and of itself, ethnic identity does not necessarily intensify internalized stigma in individuals with schizophrenia. This implies that, within the context of this population segment, the schizophrenia experience along with other factors such as psychosocial history may exert more influence on internalized stigma than ethnic identity alone.

A key finding in this study was that premorbid adjustment emerged as a significant predictor for the high and moderate stigma profiles alike. Specifically, individuals with poor premorbid adjustment were more likely to be classified into the high stigma group, with a somewhat lesser association to the Moderate stigma group. These results are in line with previous studies^(14)^, which suggested that individuals with poor psychosocial functioning prior to psychosis onset are at a heightened risk of internalizing negative stereotypes. It is plausible that people who struggled in social, educational or occupational domains before psychosis onset may have had fewer resources to combat stigma once diagnosed, rendering them more vulnerable to internalizing societal preconceptions^(37)^. These individuals may have also been more susceptible to negative social judgments, which might have contributed to greater stigma internalization. Interventions to address stigma can include Cognitive Behavioral Therapy, which challenges negative beliefs and acceptance; Commitment Therapy, which encourages accepting the disorder; and Psychoeducation programs to raise awareness and teach coping strategies^(38-40)^.

This finding emphasizes the critical role of premorbid functioning in stigma experiences and underscores the need for tailored interventions aimed at mitigating the problem. Clinical strategies should consider the unique needs of individuals with a history of poor psychosocial adjustment before psychosis onset. These patients may benefit from interventions designed to improve their social functioning and coping mechanisms, which in turn might reduce the internalization of negative societal attitudes. The significant association between premorbid adjustment and internalized stigma suggests that early psychosocial development should be a central focus, both in research and in the clinical practice.

Furthermore, the study found that severity of the symptoms, as measured by the Positive and Negative Syndrome Scale (PANSS), was not a significant predictor of stigma profiles. This is consistent with other research studies suggesting that the relationship between severity of the symptoms and self-stigma is complex and not always straightforward^(36)^. Psychosocial factors such as premorbid adjustment may play a more central role in shaping stigma experiences than severity of the psychotic symptoms themselves. This finding calls for a broader understanding of the factors influencing stigma, beyond severity of the symptoms alone.

Despite the valuable insights provided by this study, several limitations must be acknowledged. In the first place, the cross-sectional design restricts our ability to assess the stability of internalized stigma profiles over time or to determine whether the covariates identified (such as premorbid adjustment) exert a lasting impact on stigma progression. Longitudinal research studies would offer a clearer understanding of how internalized stigma evolves and how various predictors influence stigma progression over time. In particular, Latent Transition Analysis (LTA) might be a useful method to examine how people transition between stigma profiles across different treatment and recovery stages.

Another limitation is the study focus on a specific clinical setting: Chilean public Mental Health centers. While these centers provide important insights into stigma within a diverse clinical population, the findings may not be directly generalizable to other settings or population groups, especially those in different cultural contexts or healthcare systems. Future studies should explore the role of premorbid adjustment and other stigma predictors in different geographical and cultural environments to validate robustness of these findings.

## Conclusion

In conclusion, this study underscores the usefulness of Latent Class Analysis in classifying internalized stigma profiles in people with schizophrenia. The results suggest that, while belonging to some ethnic group is not a significant predictor of higher internalized stigma levels in this population segment, poor premorbid adjustment is in fact a significant predictor of the high and moderate stigma profiles alike. These findings underscore the importance of incorporating the subjects’ psychosocial history (particularly premorbid adjustment) into interventions designed to reduce stigma. Individuals with a history of poor premorbid functioning may require more targeted interventions aimed at enhancing their social functioning and coping skills, which might mitigate stigma internalization.

Future research should continue to examine the role of premorbid adjustment, severity of the symptoms and other psychosocial factors in shaping stigma progression, ideally using longitudinal designs to record the dynamic nature of this problem over time. Additionally, interventions aimed at reducing stigma in schizophrenia should be tailored to address these psychosocial factors, and future studies should explore the most effective methods for targeting internalized stigma across various subgroups within this population segment.
