# Supplementary material for: Self-stigma profiles in schizophrenia: a Latent Class Analysis approach
Source: Rev Lat Am Enfermagem. 2025 Jul 11;33:e4593. doi: 10.1590/1518-8345.7504.4593 (PMC12259217; doi:10.1590/1518-8345.7504.4593)
Supplement: Translation

# Translation: Perfis de autoestigma em pacientes com esquizofrenia: uma abordagem de Análise de Classe Latente^*^

**DOI:** 10.1590/1518-8345.7504.4594

**Authors:** Alejandra Caqueo-Urízar, Felipe Ponce-Correa, Alfonso Urzúa, Guillaume Fond, Laurent Boyer

**Affiliations:** 1 Universidad de Tarapacá, Instituto de Alta Investigación, Arica, Arica y Parinacota, Chile.; 2 Universidad de Tarapacá, Escuela de Psicología y Filosofía, Arica, Arica y Parinacota, Chile.; 3 Universidad Católica del Norte, Escuela de Psicología, Antofagasta, Antofagasta, Chile.; 4 Aix-Marseille Université, Public Health, Chronic Diseases and Quality of Life - Research Unit, Marseille, Provence-Alpes-Côte d’Azur, França.

**Keywords:** Esquizofrenia, Estigma Social, Análise de Classes Latentes, Serviços de Saúde Mental, Fatores Sociodemográficos, Escalas de Graduação Psiquiátrica

## Abstract

o objetivo deste estudo foi analisar perfis latentes de estigma internalizado e as covariáveis que preveem variações em seus níveis, considerando antecedentes como origem étnica, sexo e determinadas características clínicas relevantes, como ajuste pré-mórbido, duração da psicose não tratada e sintomas.

a Análise de Classe Latente (ACL) foi utilizada para desenhar uma solução com três perfis de estigma internalizado em uma amostra composta por 227 pacientes diagnosticados com esquizofrenia atendidos em centros públicos de saúde mental na cidade de Arica, Chile.

os resultados evidenciaram que o ajuste pré-mórbido é um preditor significativo de pertencimento a uma classe para perfis de estigma latente. Ao analisar as características sociodemográficas e, contrariamente à nossa hipótese, a origem étnica não foi um preditor relevante dos perfis de estigma internalizado.

o modelo de classificações latentes é adequado para avaliar perfis de estigma a fim de projetar intervenções futuras com focos específicos e populações de risco.

## Destaques

**(1)** Na Análise de Classe Latente identificaram-se diferentes perfis de estigma internalizado (alto, moderado e baixo) em pacientes com esquizofrenia. **(2)** A identidade étnica não é um fator de previsão significativo: contrariamente às presunções comuns, a pertença a um grupo étnico não exacerbou o estigma internalizado em indivíduos com esquizofrenia. **(3)** O ajuste pré-mórbido foi um fator-chave: o funcionamento psicossocial prejudicado antes do início da psicose foi um preditor significativo de perfis de estigma alto e moderado. **(4)** A gravidade dos sintomas foi irrelevante: a gravidade dos sintomas psicóticos não se correlacionou com o estigma internalizado, sugerindo que fatores psicossociais exercem uma influência maior. **(5)** São necessárias intervenções especificamente projetadas: pessoas com adaptação pré-mórbida precária podem se beneficiar da participação em programas que visam reduzir o estigma e focar na melhoria de suas habilidades sociais e de enfrentamento.

## Introdução

O estudo do estigma internalizado em grupos populacionais com transtornos mentais graves ganhou especial relevância nas últimas décadas^(1-2)^. Pessoas expostas a ambientes e comunidades onde o estigma de um transtorno mental está presente acabam internalizando preconceitos e estereótipos negativos que afetam sua identidade e produzem um efeito negativo em sua saúde mental e em múltiplos resultados clínicos^(3-5)^. Isso inclui sintomas depressivos^(6)^, ideação suicida^(7-8)^, diminuição da autoestima^(9-10)^, menor qualidade de vida^(11)^, fragilização dos laços sociais^(12)^ e dificuldades relacionadas ao empoderamento^(13)^, resiliência^(14)^, adesão ao tratamento^(15)^ e acesso a cuidados psiquiátricos^(16)^. Também foi encontrada uma relação entre o estigma e os efeitos colaterais dos medicamentos^(17)^.

Nos transtornos mentais graves, o aumento do nível de autoestigma reduz a capacidade de recuperação^(11)^, dificultando a remissão dos sintomas^(18)^, diminuindo a funcionalidade e a capacidade de cumprir com os papéis sociais^(19)^ e gerando efeitos negativos na subjetividade da pessoa, que acaba por perder a esperança e a sua própria autoestima^(9-10)^. Fatores como os sintomas do espectro autista na população esquizofrênica e o sexo, também exercem influência. Neste caso, as mulheres tendem a sofrer níveis mais elevados de estigma^(20)^.

Embora as consequências do autoestigma já tenham sido exploradas em profundidade, o mesmo não ocorre com seus fatores de risco e determinantes sociais. Vários estudos anteriores demonstraram que a discriminação sofrida por pessoas com esquizofrenia que pertencem a minorias étnicas ou com baixos níveis socioeconômicos aumenta o risco de estigmatização internalizada^(21)^. Por outro lado, os pacientes com psicose que não recebem tratamento há mais tempo correm maior risco de autoestigma^(22)^. Também foi estabelecido que, especialmente quando o estigma provém de membros da família, pode aumentar o limiar para o início do tratamento^(23)^, pelo que a direção desta relação e os mecanismos que a constituem ainda requerem mais desenvolvimento. Também foi descoberto que o mau funcionamento social pré-mórbido aumenta o risco de estigmatização internalizada em pacientes com esquizofrenia^(14)^. A má adaptação pré-mórbida limita severamente o desenvolvimento sociocognitivo, tanto no que diz respeito à capacidade de interpretar o mundo social quanto no que tange à presença de múltiplos estados psicológicos internos^(24)^, o que reduz a competência do paciente de lidar com significados negativos provenientes do mundo social.

Apesar do crescente corpo de literatura sobre o autoestigma, no que tange à interação dos múltiplos fatores de risco (como a discriminação, a dinâmica familiar e o funcionamento pré-mórbido) a investigação continua sendo insuficiente, particularmente nas populações latino-americanas. Nessas regiões, a escassez de profissionais de saúde mental e psiquiatras dificulta ainda mais a implementação de ferramentas padronizadas para avaliar o autoestigma. Este estudo visa identificar os fatores demográficos, psicossociais e clínicos que contribuem para níveis elevados de autoestigma em indivíduos com esquizofrenia na América Latina. A hipótese é que fatores como a etnia, o género, o funcionamento ou ajuste pré-mórbido, a duração da psicose não tratada (DUP) e a gravidade da sintomatologia psicótica seriam os preditores significativos do autoestigma nesta população. Especificamente, a etnia pode influenciar as experiências de estigma através da identidade cultural, enquanto o género pode afetar a percepção e a interiorização do estigma devido a diferenças baseadas no sexo. O ajustamento pré-mórbido, que inclui as relações sociais, as interações com os pares, o desempenho escolar, o funcionamento ocupacional e o envolvimento sócio-sexual antes do início da esquizofrenia, também pode desempenhar um papel fundamental na formação do autoestigma. Além disso, espera-se que os períodos mais longos de psicose não tratada e a sintomatologia mais grave contribuam para níveis mais elevados de autoestigma. Ao explorar estes fatores em seu contexto cultural e no âmbito dos cuidados de saúde locais, este estudo procura fornecer informações valiosas sobre o desenvolvimento e a persistência da autoestigmatização nesta população.

## Métodos

### Design do estudo

Este estudo utilizou um delineamento transversal com abordagem correlacional para investigar a associação entre estigma internalizado e vários fatores clínicos, sociodemográficos e psicossociais em pessoas diagnosticadas com esquizofrenia. A natureza transversal do estudo permitiu avaliar as relações entre as variáveis em um momento específico, fornecendo assim um retrato dos fatores que exercem alguma influência no estigma internalizado neste grupo populacional.

### Local

Este estudo foi realizado nos três centros do Serviço Público de Saúde Mental de Arica, no Chile. Os centros participantes fazem parte do Serviço de Saúde Mental do Estado. Desse modo, o atendimento prestado aos pacientes é gratuito, destacando-se que também recebem medicamentos antipsicóticos gratuitamente. O Estado garante uma cobertura de saúde que assegura, por lei, o tratamento desse transtorno psiquiátrico. O tratamento inicia-se após o primeiro surto psicótico, quando o paciente dá entrada na Unidade Psiquiátrica do Hospital Dia e, uma vez compensado, prossegue o seu tratamento em regime ambulatorial num destes três serviços, considerando a sua proximidade geográfica. Geralmente, os pacientes são supervisionados com acompanhamento mensal.

### Participantes

Os participantes foram pessoas diagnosticadas com esquizofrenia, segundo os critérios indicados na Classificação Internacional de Doenças-Revisão 10 (CID-10). Foi utilizada amostragem não probabilística devido à disponibilidade. Os participantes deviam atender aos critérios enunciados a seguir para serem incluídos no estudo.

#### Critérios de inclusão

Os sujeitos incluídos no estudo foram todos pacientes estabilizados, residentes na comunidade e diagnosticados com esquizofrenia de acordo com os critérios estabelecidos na Classificação Internacional de Doenças (CID) - Versão 10. Essas informações foram fornecidas pelo psiquiatra responsável.

#### Critérios de exclusão

Pacientes com histórico de distúrbios neurológicos (AVC, epilepsia e lesão cerebral, por exemplo) ou qualquer doença que afetasse o Sistema Nervoso Central não foram incluídos neste estudo para assegurar que todos os indivíduos pudessem participar integralmente das entrevistas. Todos os pacientes possuíam exames médicos em seus prontuários que descartavam qualquer causa orgânica que justificasse seus comportamentos psicóticos.

Como a maioria dos pacientes se encontrava estável em relação aos sintomas psicóticos, o número de indivíduos excluídos foi baixo. Além disso, a grande maioria concordou em participar.

### Variáveis e instrumentos

#### Variável de resultado

O desfecho principal do estudo foi o estigma internalizado, que foi avaliado usando a escala de Estigma Internalizado de Transtornos Mentais (*Internalized Stigma of Mental Illness*, ISMI-29)^(25)^. A escala ISMI é um questionário de autorrelato de 29 itens projetado para medir o estigma internalizado por meio de cinco subescalas: Alienação, Internalização de estereótipos, Isolamento social, Discriminação percebida e Resistência ao estigma. Pontuações totais mais altas no ISMI indicam que o estigma internalizado é mais grave. Foi utilizada a versão espanhola do ISMI^(26)^.

#### Variáveis preditoras

Escala de Síndromes Positiva e Negativa (*Positive and Negative Syndrome Scale*, PANSS) para Esquizofrenia^(27)^. A PANSS é uma escala de 30 itens utilizada para avaliar sintomas psicóticos em pessoas com esquizofrenia e é dividida em cinco fatores: Positivo, Negativo, Excitação, Depressão e Desorganização^(28)^. A PANSS foi traduzida e validada para o espanhol^(29)^.

Escala de Ajuste Pré-mórbido (*Premorbid Adjustment Scale*, PAS)^(30)^. Esta escala usa entrevistas retrospectivas para avaliar o desenvolvimento psicossocial dos pacientes antes do início da psicose, com foco em cinco domínios: Sociabilidade e isolamento, Relacionamentos com colegas, Desempenho escolar, Adaptação escolar e Ajustamento sociossexual. Pontuações PAS mais altas indicam níveis mais baixos de ajuste pré-mórbido. A escala foi adaptada para o espanhol^(31)^.

Duração da psicose não tratada (*Duration of Untreated Psychosis*, DUP)^(32)^. A DUP foi definida como o tempo decorrido desde a emergência do primeiro episódio psicótico agudo até o início do tratamento antipsicótico. A pontuação DUP foi calculada com base em informações fornecidas pelos pacientes, seus cuidadores primários e seu prontuário médico.

#### Possíveis variáveis de confusão

Certas variáveis sociodemográficas, como “origem étnica” e “sexo biológico”, foram consideradas potenciais variáveis de confusão na análise. A etnia foi avaliada por autorrelato: os participantes se autocategorizaram como pertencentes a um grupo étnico (1) ou não (2). Essa categorização reflete distinções culturais e de identidade que são relevantes para as experiências de estigma. O sexo biológico foi registrado como uma variável binária (1 = Masculino; 2 = Feminino), com base na autoidentificação relatada pelos participantes. Essas variáveis sociodemográficas foram incluídas na análise para considerar sua potencial influência no autoestigma, uma vez que tanto a etnia quanto o sexo demonstraram ter efeitos variados nas experiências relacionadas ao estigma em vários contextos de saúde mental.

Além das variáveis primárias de interesse, diversas características sociodemográficas e clínicas foram coletadas para aprimorar a descrição da amostra do estudo. Embora essas variáveis não estivessem diretamente relacionadas à hipótese, elas forneceram um contexto valioso para a compreensão do grupo populacional estudado.

A idade foi registrada como uma variável contínua; isso nos permitiu definir um perfil demográfico detalhado da amostra. O estado civil foi avaliado usando uma variável binária: os participantes indicaram se estavam em um relacionamento ou não (Sim = 1, Não = 0). Tanto a idade no primeiro episódio psicótico agudo quanto a idade do início do tratamento foram registradas como variáveis contínuas.

### Fontes de dados/Medições

Os dados primários foram coletados por meio de entrevistas estruturadas presenciais conduzidas por psicólogos treinados. Cada paciente foi avaliado individualmente em um ambiente privado em seu respectivo Centro de Saúde Mental. As escalas ISMI-29, PAS e PANSS foram aplicadas como parte do processo de entrevista. Igualmente, informações adicionais sobre DUP foram coletadas de registros médicos, autorrelatos de pacientes e dados de cuidadores. Todos os instrumentos de medição utilizados no estudo são ferramentas comprovadas e foram validados para uso com populações de língua espanhola. Esses instrumentos também demonstraram bons níveis de confiabilidade e validade em pesquisas anteriores.

### Viés

Várias medidas foram tomadas ao longo do estudo para minimizar potenciais fontes de viés. O viés de seleção foi atenuado pela inclusão de participantes de todos os três centros públicos de saúde mental, o que permitiu uma maior variedade de experiências clínicas. Entretanto, o método de amostragem não probabilística (conveniência) limita a capacidade de generalizar os resultados para a população geral de pessoas com esquizofrenia. O viés de medição foi minimizado pela padronização dos procedimentos de entrevista: as avaliações foram conduzidas por dois psicólogos devidamente treinados, sob a supervisão do pesquisador principal. Para abordar o viés de memória, protocolos de entrevista estruturados foram utilizados a fim de orientar as lembranças dos participantes. Por outra parte, os prontuários médicos foram consultados para obter informações corroborativas quando disponíveis.

### Tamanho da amostra

Um total de 227 pacientes diagnosticados com esquizofrenia foram recrutados. O tamanho da amostra foi determinado com base na viabilidade de recrutamento de pacientes nos três centros e no número de participantes elegíveis que compareceram às consultas regulares de acompanhamento. Esse tamanho amostral foi considerado adequado para alcançar poder estatístico suficiente para as análises planejadas, que incluíram Análise de Classe Latente (ACL) e regressão logística multinomial, com o objetivo de detectar associações significativas entre as variáveis preditoras e o estigma internalizado.

Durante um período de três meses, pacientes que compareceram às consultas mensais de acompanhamento em centros públicos e primários de tratamento ambulatorial para esquizofrenia foram convidados a participar. Dois psicólogos (que faziam parte da equipe de pesquisa e atuavam sob a supervisão do pesquisador principal) realizaram as avaliações nos respectivos Centros de Saúde Mental de cada paciente. As sessões de avaliação tiveram uma duração aproximada de 40 a 60 minutos.

Todos os participantes prestaram consentimento informado por escrito antes do início da coleta de dados. Os objetivos do estudo foram explicados a todos os participantes, assim como a natureza voluntária de sua participação.

### Métodos estatísticos

Os dados foram analisados por meio da Análise de Classe Latente (ACL)^(33)^, um método estatístico desenvolvido para identificar diferentes subgrupos ou perfis dentro de um segmento populacional específico com base nos padrões de resposta detectados no ISMI-29. O número de classes latentes foi determinado utilizando uma combinação de índices de ajuste do modelo, como o Critério de Informação Bayesiano (*Bayesian Information Criterion*, BIC), o Critério de Informação de Akaike (*Akaike Information Criterion*, AIC) e o Critério de Informação Bayesiano ajustado (*adjusted Bayesian Information Criterion*, aBIC)^(33-34)^. Valores mais baixos nesses índices indicam modelos de melhor ajuste. Os testes de razão de verossimilhança ajustada de Lo-Mendell-Rubin e *bootstrap* também foram usados para avaliar o ajuste do modelo.

A regressão logística multinomial foi usada para examinar a associação entre perfis de estigma internalizados e diversas variáveis preditoras, como etnia, sexo, ajuste pré-mórbido, DUP e gravidade dos sintomas psicóticos. Essa abordagem permitiu que vários preditores e potenciais fatores de confusão fossem analisados simultaneamente. Várias análises de subgrupos também foram conduzidas para investigar se as relações entre preditores e perfis de estigma diferiam de acordo com as características demográficas^(34)^.

Os dados faltantes foram tratados usando imputação múltipla. Igualmente, várias análises de sensibilidade foram conduzidas para avaliar a robustez dos resultados em relação a diferentes pressupostos sobre os dados faltantes. Todas as análises foram executadas no Mplus (versão 8.5)^(35-36)^.

### Considerações éticas

O estudo foi aprovado pelo Comitê de Ética da Universidad de Tarapacá (18/2009) e pelo Serviço Nacional de Saúde do Chile. O consentimento informado foi obtido tanto dos pacientes quanto de seus cuidadores primários. O estudo enfatizou a natureza voluntária da participação e nenhuma compensação financeira foi oferecida aos participantes.

## Resultados

Os resultados correspondentes à análise descritiva da amostra mostraram que a média de idade foi de 41,1 anos [Desvio-Padrão (DP)=16,34], com 129 (56,8%) pacientes do sexo masculino, 181 (79,7%) sem companheiro e 86 (37,9%) que se autodeclararam pertencentes à etnia aimará. A idade dos participantes no momento do primeiro episódio psicótico agudo e no início do tratamento foi de 21,4 anos (DP=8,4) e 25 anos (DP=8,9), respectivamente.

O fluxograma (Figura 1) apresenta uma representação visual clara do processo de seleção de participantes, começando com um total de 423 candidatos potenciais nos três centros de tratamento. Após a triagem de elegibilidade, 240 indivíduos foram avaliados para inclusão no estudo. Dez indivíduos foram excluídos devido a sintomas clinicamente significativos que os impediram de participar plenamente do estudo. Três pessoas se recusaram a participar após receber o convite. Portanto, a amostra incluída para análise foi composta por 227 indivíduos que atenderam aos critérios de inclusão e prestaram consentimento informado. O fluxograma ilustra esse processo passo a passo, garantindo assim transparência no recrutamento e inclusão de participantes.

**Figura 1 -:** Fluxograma ilustrando o processo de seleção e inclusão de participantes

Os resultados da ACL confirmaram que o modelo de três classes apresentou os melhores indicadores de ajuste relativo [AIC=1976,884; aBIC=1982,509; aLMR=89,914; Regressão Linear Bayesiana (BLR)=92,676]. A entropia correspondente ao modelo de três classes atingiu um nível adequado (0,82).

A Tabela 1 apresenta as pontuações médias correspondentes ao estigma internalizado em cada uma das dimensões do ISMI-29 e a distribuição das covariáveis. Entre os pacientes com perfis latentes de estigma alto, 50% eram homens e 47,9% se identificaram como pertencentes a algum grupo étnico. Também apresentaram a pior média de ajuste pré-mórbido (13,75), a maior duração de psicose não tratada em anos (3,85) e os piores sintomas na amostra (63,45). Por outro lado, o perfil latente de Estigma Moderado foi composto majoritariamente por homens (62,4%) e pessoas que se identificaram como pertencentes a algum grupo étnico (58,4%). Também apresentaram nível intermediário de ajuste pré-mórbido (10,82), psicose não tratada em anos (1,88) e sintomas (61,83). Por fim, o perfil latente de Estigma Baixo foi composto majoritariamente por homens (61,5%) e pessoas que se identificaram como pertencentes a algum grupo étnico (59,6%). Apresentou os melhores níveis de ajuste pré-mórbido (9,45), duração de psicose não tratada em anos (1,58) e sintomas (59,54).

**Tabela 1 -:** Estatísticas descritivas correspondentes às variáveis clínicas. Arica, Arica e Parinacota, Chile, 2023

Valor médio (DP*)					
**ISMI** ^†^		**Amostra total**	**Estigma alto**	**Estigma moderado**	**Estigma baixo**
Alienação		13,34 (4,22)	2,69 (0,06)	2.218 (0,06)	1.359 (0,06)
Percepção de discriminação		11,26 (3,68)	2,87 (0,05)	2.131 (0,07)	1.363 (0,07)
Autoestigma		14,60 (4,71)	2,66 (0,04)	1.974 (0,07)	1.243 (0,06)
Resistência ao estigma		12,80 (2,92)	2,33 (0,06)	2.659 (0,06)	2.867 (0,10)
Evitação social		13h45 (5h06)	2,84 (0,07)	2.172 (0,11)	1.270 (0,07)
(DP*)/Frequência (%)					
**Covariáveis**		**Amostra total (n=227)**	**Estigma alto (n=98)**	**Estigma moderado (n=77)**	**Estigma baixo (n=52)**
Sexo	Masculino	129 (56,8%)	49 (50%)	48 (62,4%)	32 (61,5%)
	Feminino	98 (43,2%)	49 (50%)	29 (37,6%)	20 (38,5%)
Grupo étnico	Sim	123 (54,2%)	47 (47,9%)	45 (58,4%)	31 (59,6%)
	Não	104 (45,8%)	51 (52,1%)	32 (41,6%)	21 (40,4%)
Ajuste pré-mórbido		11,52 (4,45)	13,75 (4,30)	10,82 (4,01)	9,45 (3,70)
Duração da psicose não tratada		2,46 (6,36)	3,85 (8,76)	1,88 (5,17)	1,58 (3,11)
Sintomas		61,80 (18,91)	63,45 (16,19)	61,83 (21,64)	59,5 (19,43)

*DP = Desvio-Padrão; ^†^ISMI = *Internalized Stigma of Mental Illness*

A Figura 2 mostra a distribuição dos perfis latentes por meio de um *boxplot* para cada uma das classes e dimensões do ISMI-29. Pode-se afirmar que os indivíduos com tendências autoestigmatizantes significativas e menor resistência ao estigma, mais propensos a concordar com estereótipos negativos sobre esquizofrenia, sofreram mais discriminação e apresentam maiores níveis de isolamento ou evitação social (n=98) foram agrupados em (C1). Por outro lado, pessoas com níveis médios em todas as dimensões do ISMI-29 foram incluídas em** **(C2)** **(n=77). Por sua vez, em (C3) encontramos participantes com maior resistência ao estigma e menores tendências autoestigmatizantes, capazes de ter uma visão positiva de si e da doença, com pouca percepção da discriminação e maior participação social (n=52).

**Figura 2 -:** Valores médios correspondentes aos perfis latentes de estigma internalizado segundo as dimensões do ISMI-29

*C3 = Estigma baixo; ^†^C2 = Estigma moderado; ^‡^C1 = Estigma alto

A Tabela 2 apresenta os resultados da regressão logística multinomial, examinando a associação entre as covariáveis e a filiação de classe latente para os perfis de estigma internalizado. O perfil de estigma baixo é usado como grupo de referência para comparação.

**Tabela 2 -:** Resultados da regressão logística multinomial para covariáveis de classe latentes. Arica, Arica e Parinacota, Chile, 2023

**Grupo de referência**	**Grupo de comparação**	**Grupo étnico**	**Sexo**	**Duração da psicose não tratada**	**Ajuste pré-mórbido**	**Sintomas**
OR* (IC ^†^ )						
Estigma alto	Estigma moderado	1,09(0,76; 1,56)	0,34(0,04; 2,92)	0,91(0,79; 1,06)	0,87(0,71; 1,06)	0,94(0,88; 1,01)
	Estigma baixo	0,88(0,65; 1,19)	0,33(0,06; 1,71)	0,98(0,89; 1,08)	0,70(0,58; 0,85) ^‡^	0,98(0,93; 1,04)
Estigma baixo	Estigma moderado	1,23(0,86; 1,76)	1,04(0,22; 4,8)	0,93(0,80; 1,08)	1,23(1,00; 1,5) ^‡^	0,95(0,90; 1,01)

Nota: O perfil de estigma baixo foi usado como grupo de referência para comparação; *OR = *Odds Ratio*; ^†^IC = Intervalo de confiança; ^‡^Significância estatística

Conforme apontado na Tabela 2, o ajuste pré-mórbido foi o único preditor significativo de pertencimento nos perfis de estigma alto e baixo. Especificamente, o ajuste pré-mórbido prejudicado (OR=0,70 para estigma alto e OR=1,23 para estigma moderado) aumenta a probabilidade de uma pessoa ser classificada nos perfis moderado ou alto de estigma internalizado, em comparação ao grupo de estigma baixo.

Isso sugere que indivíduos com pior funcionamento psicossocial antes do início da psicose são mais predispostos a sofrer níveis mais altos de estigma internalizado, em comparação com aqueles com melhor ajuste pré-mórbido, que são mais propensos a ser categorizados no grupo de estigma baixo.

## Discussão

Na Análise de Classe Latente (ACL) realizada neste estudo, foram identificados três perfis diferentes de estigma internalizado em pessoas diagnosticadas com esquizofrenia, a saber: alto, moderado e baixo. A classificação de três perfis provou ser um modelo eficaz para categorizar participantes com base em suas pontuações de estigma latente. Contrariando a nossa hipótese, a origem étnica não surgiu como fator preditor significativo de pertencimento ao perfil de estigma alto. Esse achado questiona certas presunções predominantes na literatura sobre estigma. Ainda que pesquisas anteriores tenham frequentemente enfatizado a relação entre pertencer a um grupo étnico e a intensificação do estigma^(1)^ e contradizendo a nossa hipótese, sofrer de esquizofrenia e pertencer a um segmento social vulnerável como um grupo étnico^(13)^ provavelmente não aumenta os níveis de estigma, rejeitando assim a combinação de condições proposta como hipótese. Esses resultados sugerem que a identidade étnica por si só não intensifica necessariamente o estigma internalizado em pessoas com esquizofrenia. Isso implica que, no contexto desse segmento da população, sofrer de esquizofrenia e apresentar outros fatores, como antecedentes psicossociais, em conjunto, pode exercer uma influência maior no estigma internalizado do que a identidade étnica isoladamente.

Um achado importante deste estudo foi o fato de que o ajuste pré-mórbido surgiu como um preditor significativo para os perfis de estigma alto e moderado. Especificamente, indivíduos com ajuste pré-mórbido ruim tinham maior probabilidade de serem categorizados no grupo de estigma alto, com uma associação ligeiramente mais fraca com o grupo de estigma moderado. Esses resultados são consistentes com os de estudos anteriores^(14)^, que sugerem que pessoas com funcionamento psicossocial prejudicado antes do início da psicose correm maior risco de internalizar estereótipos negativos. É plausível que as pessoas que vivenciaram dificuldades nos domínios social, educacional ou ocupacional antes do início da psicose tivessem menos recursos para combater o estigma uma vez diagnosticadas, sendo mais vulneráveis à internalização de preconceitos sociais^(37)^. Esses indivíduos também podem ter sido mais suscetíveis a julgamentos sociais negativos, o que pode ter contribuído para uma maior internalização de seu estigma. As intervenções para lidar com o estigma incluem: Terapia cognitivo-comportamental, que desafia crenças negativas e aceitação; Terapia de Compromisso, que estimula a aceitação do transtorno; e programas de psicoeducação para conscientizar e ensinar estratégias de enfrentamento^(38-40)^.

Essa descoberta enfatiza o papel crítico do funcionamento pré-mórbido em termos de experiências relacionadas ao estigma e destaca a necessidade de projetar intervenções personalizadas destinadas a mitigar o problema. As estratégias clínicas devem considerar as necessidades específicas de indivíduos com histórico de funcionamento psicossocial prejudicado antes do início da psicose. Esses pacientes podem se beneficiar da participação em intervenções projetadas para melhorar seu funcionamento social e mecanismos de enfrentamento, o que, por sua vez, pode reduzir a internalização de atitudes negativas em relação à sociedade. A associação significativa entre ajuste pré-mórbido e estigma internalizado sugere que o desenvolvimento psicossocial precoce deve ser um foco central de atenção tanto na pesquisa quanto na prática clínica.

Além disso, o estudo descobriu que a gravidade dos sintomas, medida pela Escala de Síndromes Positiva e Negativa (PANSS), não foi um preditor significativo de perfis de estigma. Isto é consistente com outros estudos de investigação que sugerem que a relação entre a gravidade dos sintomas e o autoestigma é complexa e nem sempre directa^(36)^. Vários fatores psicossociais, como o ajuste pré-mórbido, podem desempenhar um papel mais central na formação de experiências de estigma do que a gravidade dos sintomas psicóticos em si. Essa descoberta exige uma melhor compreensão dos fatores que influenciam o estigma além da gravidade dos sintomas.

Apesar dos detalhes valiosos fornecidos por este estudo, diversas limitações precisam ser reconhecidas. Primeiro, o desenho transversal restringe nossa capacidade de avaliar a estabilidade dos perfis de estigma internalizados ao longo do tempo ou de determinar se as covariáveis identificadas (ajuste pré-mórbido, por exemplo) exercem algum efeito duradouro na evolução do estigma. A realização de estudos de pesquisa longitudinais proporcionaria uma compreensão mais clara de como o estigma internalizado progride e das diferentes influências exercidas por vários preditores na progressão do estigma ao longo do tempo. Em particular, uma Análise de Transição Latente (ATL) pode ser um método útil para examinar o modo como as pessoas passam de um perfil de estigma para outro durante diferentes estágios de seu tratamento e recuperação.

Outra limitação é o fato de o estudo ter se concentrado em um ambiente clínico específico: Centros Públicos de Saúde Mental no Chile. Embora esses centros forneçam dados importantes sobre o estigma em uma população clínica diversificada, não é possível generalizar diretamente os achados para outros cenários ou grupos populacionais, especialmente aqueles de diferentes contextos culturais ou sistemas de saúde. Estudos futuros devem investigar o papel do ajuste pré-mórbido e outros preditores de estigma em diferentes contextos geográficos e culturais para validar a robustez dessas descobertas.

## Conclusão

Concluindo, este estudo destaca a utilidade da Análise de Classe Latente na classificação de perfis de estigma internalizado em pessoas com esquizofrenia. Os resultados sugerem que, embora pertencer a um grupo étnico não seja um preditor significativo de níveis mais altos de estigma internalizado nesse segmento da população, o ajuste pré-mórbido prejudicado prevê significativamente os perfis de estigma alto e moderado. Essas descobertas destacam a importância de incorporar o contexto psicossocial dos sujeitos (particularmente seu ajuste pré-mórbido) em intervenções projetadas para reduzir o estigma. Indivíduos com histórico de mau funcionamento pré-mórbido podem precisar de intervenções mais personalizadas, visando melhorar seu funcionamento social e habilidades de enfrentamento, o que pode reduzir a internalização do estigma.

Pesquisas futuras devem prosseguir a análise do papel do ajuste pré-mórbido, da gravidade dos sintomas e de outros fatores psicossociais no desenvolvimento do estigma. O ideal seria usar modelos longitudinais para capturar a natureza dinâmica desse problema ao longo do tempo. Além disso, intervenções personalizadas que visam reduzir o estigma na esquizofrenia devem ser adaptadas para abordar esses fatores psicossociais, e estudos futuros devem explorar os métodos mais eficazes para tratar o estigma internalizado em vários subgrupos dentro desse segmento populacional.

# Translation: Perfiles de autoestigma en pacientes con esquizofrenia: enfoque basado en Análisis de Clases Latentes*

**DOI:** 10.1590/1518-8345.7504.4592

**Authors:** Alejandra Caqueo-Urízar, Felipe Ponce-Correa, Alfonso Urzúa, Guillaume Fond, Laurent Boyer

**Affiliations:** 1 Universidad de Tarapacá, Instituto de Alta Investigación, Arica, Arica y Parinacota, Chile.; 2 Universidad de Tarapacá, Escuela de Psicología y Filosofía, Arica, Arica y Parinacota, Chile.; 3 Universidad Católica del Norte, Escuela de Psicología, Antofagasta, Antofagasta, Chile.; 4 Aix-Marseille Université, Public Health, Chronic Diseases and Quality of Life - Research Unit, Marseille, Provence-Alpes-Côte d’Azur, Francia.

**Keywords:** Esquizofrenia, Estigma Social, Análisis de Clases Latentes, Servicios de Salud Mental, Factores Sociodemográficos, Escalas de Valoración Psiquiátrica

## Abstract

el propósito de este estudio fue analizar los perfiles latentes de estigma internalizado y las covariables que predicen variaciones en sus niveles considerando antecedentes como origen étnico, sexo y ciertas características clínicas relevantes como ajuste premórbido, Duración de psicosis no tratada y síntomas.

se utilizó Análisis de Clases Latentes (ACL) para diseñar una solución con tres perfiles de estigma internalizado en una muestra compuesta por 227 pacientes con diagnóstico de esquizofrenia atendidos en los centros públicos de Salud mental de la ciudad de Arica, Chile.

los resultados demostraron que el ajuste premórbido es un predictor significativo de pertenencia a una clase para los perfiles de estigma latente. Al analizar las características sociodemográficas y contrariamente a nuestra hipótesis, el origen étnico no fue un predictor relevante de perfiles de estigma internalizado.

el modelo de clasificaciones latentes es apto para evaluar perfiles de estigma con el fin de diseñar futuras intervenciones en focos y poblaciones en riesgo específicos.

## Destacados

**(1)** En el Análisis de Clases Latentes se detectaron distintos perfiles de estigma internalizado (alto, moderado y bajo) en pacientes con esquizofrenia. **(2)** La identidad étnica no fue un predictor significativo: contrariamente a presunciones comunes, pertenecer a un grupo étnico en particular no intensificó el estigma internalizado en personas que sufren esquizofrenia. **(3)** El ajuste premórbido fue un factor clave: un funcionamiento psicosocial deficiente antes de que se desencadenara la psicosis fue un predictor significativo de perfiles de estigma alto y moderado. **(4)** La gravedad de los síntomas fue irrelevante: la gravedad de los síntomas psicóticos no presentó correlación alguna con el estigma internalizado, lo que sugiere que los factores psicosociales ejercen mayor influencia. **(5)** Se requieren intervenciones específicamente diseñadas: las personas con ajuste premórbido deficiente podrían resultar beneficiadas al participar en programas dirigidos a reducir el estigma y enfocados en mejorar sus habilidades sociales y de afrontamiento.

## Introducción

El estudio del estigma internalizado en grupos poblacionales con trastornos mentales graves ha cobrado especial relevancia en las últimas décadas^(1-2)^. Las personas expuestas a entornos y comunidades donde el estigma de algún trastorno mental está presente terminan internalizando prejuicios y estereotipos negativos que afectan su identidad y ejercen un efecto negativo sobre su salud mental y múltiples desenlaces clínicos^(3-5)^; eso incluye síntomas depresivos^(6)^, ideación suicida^(7-8)^, autoestima disminuida^(9-10)^, menor calidad de vida^(11)^, vínculos sociales empobrecidos^(12)^, y dificultades relacionadas con el empoderamiento^(13)^, la resiliencia^(14)^, la adhesión al tratamiento^(15)^ y el acceso a atención psiquiátrica^(16)^. También se encontró una relación entre estigma y efectos secundarios de medicamentos^(17)^.

En trastornos mentales graves, el aumento en el nivel de autoestigma reduce la capacidad de recuperación^(11)^, dificultando así la remisión de los síntomas^(18)^, disminuyendo la funcionalidad y la capacidad para mantener roles sociales^(19)^ y ejerciendo efectos negativos sobre la subjetividad de una persona, que eventualmente pierde la esperanza y su propia autoestima^(9-10)^. Otros factores como los síntomas del espectro autista en la población esquizofrénica y el sexo también son influyentes; en este caso, las mujeres tienden a padecer niveles de estigma más elevados^(20)^.

Aunque las consecuencias del autoestigma ya se han explorado en profundidad, no es el caso con sus factores de riesgo y determinantes sociales. Diversos estudios previos han demostrado que la discriminación sufrida por las personas con esquizofrenia que pertenecen a minorías étnicas o con niveles socioeconómicos bajos aumenta el riesgo de estigmatización internalizada^(21)^. Por otra parte, los pacientes con psicosis no sometidos a tratamiento durante períodos más prolongados corren mayor riesgo de autoestigma^(22)^. También se ha establecido que el estigma puede elevar el umbral para iniciar un tratamiento, especialmente cuando proviene de los familiares^(23)^; por lo tanto, la dirección de esta relación y el mecanismo que la constituye aún requieren mayor desarrollo. También se descubrió que un funcionamiento social premórbido deficiente aumenta el riesgo de estigmatización internalizada en pacientes con esquizofrenia^(14)^. Una adaptación premórbida deficiente limita seriamente el desarrollo sociocognitivo, tanto en la capacidad para interpretar el mundo social como en materia de diversos estados psicológicos internos^(24)^, lo que reduce la capacidad de un paciente para lidiar con los significados negativos que provienen del mundo social.

Pese al creciente volumen de literatura sobre autoestigma, el entrelazamiento de múltiples factores de riesgo (discriminación, dinámica familiar y funcionamiento premórbido, por ejemplo) todavía no se han investigado lo suficiente, particularmente en poblaciones de América Latina. En estas regiones, la escasa cantidad de psiquiatras y de profesionales especializados en Salud mental complica aún más la implementación de herramientas estandarizadas para evaluar el autoestigma. El propósito de este estudio es identificar los factores demográficos, psicosociales y clínicos que contribuyen a generar niveles de autoestigma elevados en personas que padecen esquizofrenia en América Latina. Nuestra hipótesis es que factores como origen étnico, sexo, ajuste premórbido, Duración de psicosis no tratada (*Duration of Untreated Psychosis*, DUP) y gravedad de los síntomas serían predictores significativos del autoestigma en este grupo poblacional. Específicamente, el origen étnico podría influenciar experiencias de estigma a través de la identidad cultural mientras que el sexo podría afectar la percepción e internalización del estigma a raíz de diferencias basadas en el género. El ajuste premórbido, que incluye vínculos sociales, interacciones entre pares, rendimiento académico, funcionamiento ocupacional y actividad sociosexual antes de desencadenarse la esquizofrenia, también pueden ser factores clave en el desarrollo del autoestigma. Además, se intuye que una duración de psicosis no tratada más prolongada y síntomas graves contribuirían a elevar los niveles de autoestigma. Como explora esos factores en el contexto de ambientes culturales y atención de la salud locales, este estudio procura proporcionar detalles valiosos sobre el desarrollo y la persistencia de la autoestigmatización en este segmento de la población.

## Métodos

### Diseño del estudio

En este estudio se empleó un diseño transversal con enfoque correlacional a fin de investigar la asociación entre estigma internalizado y diversos factores clínicos, sociodemográficos y psicosociales en personas con diagnóstico de esquizofrenia. El carácter transversal del estudio permitió evaluar relaciones entre variables en un momento puntual, proporcionando así una instantánea de los factores que ejercen alguna influencia sobre el estigma internalizado en este grupo poblacional.

### Lugar

Este estudio se desarrolló en los 3 centros del Servicio Público de Salud Mental de Arica, Chile. Los centros participantes forman parte del servicio de Salud mental del Estado; por consiguiente, la asistencia provista a los pacientes es gratuita, además de que se les proporcionan fármacos antipsicóticos sin cargo. El Estado tiene un plan médico que cubre la atención de este trastorno psiquiátrico por ley. El tratamiento se inicia después del primer brote psicótico, cuando el paciente ingresa a la Unidad Psiquiátrica del Hospital Día y, una vez compensado, continúa su tratamiento con régimen ambulatorio en alguno de estos tres servicios, dependiendo de su proximidad geográfica. Los pacientes suelen ser sometidos a un seguimiento mensual.

### Participantes

Los participantes fueron personas con diagnóstico de esquizofrenia, conforme a los criterios indicados en la Clasificación Internacional de Enfermedades-Revisión 10 (CIE-10). Se utilizó muestreo no probabilístico por disponibilidad. Los participantes debían cumplir los siguientes criterios para ser incluidos en el estudio.

#### Criterios de inclusión

Los sujetos incluidos en el estudio fueron todos los pacientes estabilizados que viven en la comunidad y con diagnóstico de esquizofrenia de acuerdo con los criterios establecidos en la Clasificación Internacional de Enfermedades (CIE)-Versión 10. Esta información fue proporcionada por el psiquiatra a cargo.

#### Criterios de exclusión

Los pacientes con antecedentes de trastornos neurológicos (accidente cerebrovascular, epilepsia y lesiones cerebrales, por ejemplo) o de cualquier enfermedad que afecte al Sistema Nervioso Central no fueron incluidos en este estudio para garantizar que todos los sujetos fueran capaces de participar plenamente en las entrevistas. Todos los pacientes tienen exámenes médicos en sus historias clínicas que descartan cualquier causa orgánica que justifique sus conductas psicóticas.

Como gran parte de los pacientes se encontraba estable en relación con sus síntomas psicóticos, la cantidad de sujetos excluidos fue baja; además, la inmensa mayoría aceptó participar.

### Variables e instrumentos

#### Variable de resultado

El resultado principal del estudio fue el estigma internalizado, que se evaluó utilizando la escala de Estigma internalizado de enfermedades mentales (*Internalized Stigma of Mental Illness*, ISMI-29)^(25)^. La escala ISMI es un cuestionario de autoinforme con 29 ítems diseñado para medir el estigma internalizado por medio de cinco subescalas: Alienación, Internalización de estereotipos, Aislamiento social, Discriminación percibida y Resistencia al estigma. Puntuaciones totales más altas en ISMI indican que el estigma internalizado es más grave. Se utilizó la versión de ISMI en español^(26)^.

#### Variables predictoras

Escala de síndromes positivos y negativos (*Positive and Negative Syndrome Scale*, PANSS) para la Esquizofrenia^(27)^. PANSS es una escala con 30 ítems que se utiliza para evaluar síntomas psicóticos en personas con esquizofrenia y está dividida en cinco factores: Positivos, Negativos, Excitación, Depresión y Desorganización^(28)^. PANSS se tradujo y validó al español^(29)^.

Escala de ajuste premórbido (*Premorbid Adjustment Scale*, PAS)^(30)^. Esta escala recurre a entrevistas retrospectivas para evaluar el desarrollo psicosocial de los pacientes antes de desencadenarse la psicosis, enfocándose en cinco dominios: Sociabilidad y aislamiento, Relaciones de compañerismo, Rendimiento escolar, Adaptación escolar y Ajuste sociosexual. Puntuaciones PAS más altas indican niveles de ajuste premórbido más bajos. La escala se adaptó al español^(31)^.

Duración de psicosis no tratada (*Duration of Untreated Psychosis*, DUP)^(32)^: DUP se definió como como el tiempo transcurrido desde la aparición del primer episodio psicótico agudo hasta el inicio del tratamiento antipsicótico. La puntuación DUP se calculó sobre la base de información proporcionada por los pacientes, sus principales cuidadores y las historias clínicas.

#### Variables de confusión potenciales

Ciertas variables sociodemográficas como “origen étnico” y “sexo biológico” se consideraron variables de confusión potenciales en el análisis. El origen étnico se evaluó mediante autoinforme: los participantes se autocategorizaron como pertenecientes a algún grupo étnico (1) o no (2). Esta categorización refleja distinciones culturales y basadas en la identidad que resultan relevantes para las experiencias de estigma. El sexo biológico se registró como variable binaria (1 = Masculino; 2 = Femenino), en función de la autoidentificación señalada por los participantes. Estas variables sociodemográficas se incluyeron en el análisis para dar cuenta de su posible influencia sobre el autoestigma, puesto que se ha demostrado que tanto el origen étnico como el sexo ejercen variados efectos sobre experiencias relacionadas con el estigma en diversos contextos de salud mental.

Además de las variables de interés primarias, se recolectaron varias características sociodemográficas y clínicas para acrecentar la descripción de la muestra del estudio. Si bien estas variables no estuvieron directamente relacionadas con la hipótesis, proporcionaron un contexto valioso para comprender al grupo poblacional estudiado.

La edad se registró como variable continua; eso permitió definir un perfil demográfico detallado de la muestra. La situación de pareja se evaluó utilizando una variable binaria: los participantes indicaron si estaban en una relación o no (Sí =1, No = 0). Tanto la edad al momento del primer episodio psicótico agudo como la edad al inicio del tratamiento se registraron como variables continuas.

### Fuentes de datos/Mediciones

Los datos primarios se recolectaron por medio de entrevistas presenciales estructuradas a cargo de psicólogos debidamente capacitados. Se evaluó individualmente a cada paciente en un ambiente privado de su centro de Salud mental respectivo. Se aplicaron las escalas ISMI-29, PAS y PANSS como parte del proceso de las entrevistas; además, se reunió información adicional sobre DUP de las historias clínicas, los autoinformes de los pacientes y los datos aportados por los cuidadores. Todos los instrumentos de medición empleados en el estudio son herramientas comprobadas y los mismos han sido validados para ser usados con poblaciones de habla hispana. También han demostrado buenos niveles de fiabilidad y validez en estudios de investigación previos.

### Sesgo

Se adoptaron varias medidas durante la totalidad del estudio para reducir al mínimo posibles fuentes de sesgo. El sesgo de selección se mitigó incluyendo participantes de los tres centros públicos de salud mental, lo que permitió contar con mayor variedad de experiencias clínicas. Sin embargo, el método de muestreo no probabilístico (por conveniencia) limita la capacidad de generalizar los hallazgos a la población general de personas con esquizofrenia. El sesgo de medición se redujo al mínimo estandarizando los procedimientos de las entrevistas: las evaluaciones estuvieron a cargo de dos psicólogos debidamente capacitados bajo la supervisión del investigador principal. A fin de abordar el sesgo de memoria, se utilizaron protocolos de entrevistas estructuradas para orientar los recuerdos de los participantes; además, se consultaron las historias clínicas para obtener información corroborativa cuando se encontrara disponible.

### Tamaño del estudio

Se reclutó a un total de 227 pacientes con diagnóstico de esquizofrenia. El tamaño de la muestra se determinó en función de la viabilidad para reclutar pacientes en los tres centros y de la cantidad de participantes elegibles que asistían a consultas periódicas de seguimiento. Este tamaño de la muestra se consideró adecuado a fin de alcanzar suficiente poder estadístico para los análisis planificados, que incluyeron Análisis de Clases Latentes (ACL) y regresión logística multinomial, con el objetivo de detectar asociaciones significativas entre las variables predictoras y el estigma internalizado.

Durante un período de tres meses, se invitó a participar a los pacientes que acudían a sus consultas mensuales de seguimiento en los centros públicos y primarios de tratamiento ambulatorio para la esquizofrenia. Dos psicólogos (que forman parte del equipo de investigación y actuaron bajo la supervisión del investigador principal) llevaron a cabo las evaluaciones en los centros de salud mental respectivos de cada paciente. Las sesiones evaluatorias duraron entre 40 y 60 minutos.

Todos los participantes proporcionaron su consentimiento informado por escrito antes de iniciarse la recolección de datos. Se explicaron los objetivos del estudio a todos los participantes, al igual que el carácter voluntario de su participación.

### Métodos estadísticos

Los datos se analizaron utilizando Análisis de Clases Latentes (ACL)^(33)^, que es un método estadístico diseñado para identificar diferentes subgrupos o perfiles dentro de un segmento poblacional específico en función de los patrones de respuesta detectados en ISMI-29. La cantidad de clases latentes se determinó utilizando una combinación de índices de ajuste de modelos, como Criterio de Información Bayesiano (*Bayesian Information Criterion*, BIC), Criterio de Información de Akaike (*Akaike Information Criterion*, AIC) y Criterio de Información Bayesiano ajustado (*adjusted Bayesian Information Criterion*, aBIC)^(33-34)^. Valores más bajos en estos índices indican modelos con mejor ajuste. También se emplearon las pruebas de razón de verosimilitud ajustada de Lo-Mendell-Rubin y *bootstrap* para evaluar el ajuste de los modelos.

Se utilizó regresión logística multinomial para examinar la asociación entre perfiles de estigma internalizado y diversas variables predictoras como origen étnico, sexo, ajuste premórbido, DUP y gravedad de los síntomas psicóticos. Este enfoque permitió analizar múltiples predictores y variables de confusión potenciales simultáneamente. También se realizaron diversos análisis de subgrupos para investigar si las relaciones entre los predictores y los perfiles de estigma diferían según las características demográficas^(34)^.

Los datos faltantes se abordaron utilizando imputación múltiple; además, se llevaron a cabo diversos análisis de sensibilidad para evaluar la solidez de los resultados conforme a distintas presunciones en relación a los datos faltantes. Todos los análisis se ejecutaron en *Mplus*(Versión 8.5)^(35-36)^.

### Consideraciones éticas

El estudio contó con la aprobación del Comité de Ética de la Universidad de Tarapacá (18/2009) y del Servicio Nacional de Salud de Chile. Se obtuvo el consentimiento informado tanto de los pacientes como de sus principales cuidadores. En el estudio se enfatizó el carácter voluntario de su participación y no se ofreció ninguna compensación financiera a los participantes.

## Resultados

Los resultados correspondientes al análisis descriptivo de la muestra demostraron que la media de edad fue 41,1 años [Desviación Estándar (DE)=16,34], con 129 (56,8%) pacientes del sexo masculino, 181 (79,7%) sin pareja y 86 (37,9%) que autoinformaron pertenecer al grupo étnico Aymara. La edad de los participantes al momento del primer episodio psicótico agudo y al iniciarse el tratamiento fue 21,4 años (DE=8.4) y 25 años (DE=8.9), respectivamente.

En el diagrama de flujo (Figura 1) se proporciona una clara representación visual del proceso para seleccionar a los participantes, comenzando con una cantidad total de 423 posibles candidatos entre los tres centros de tratamiento. Después del cribado para determinar la elegibilidad, se evaluó a 240 personas para ser incluidas en el estudio. Se excluyó a diez sujetos debido a síntomas clínicamente significativos que les impedían participar plenamente en el estudio. Tres personas se rehusaron a participar después de recibir la invitación. Por lo tanto, la muestra que se incluyó para el análisis estuvo conformada por 227 individuos que cumplieron los criterios de inclusión y proporcionaron su consentimiento informado. En el diagrama de flujo se ilustra este proceso paso a paso, garantizando así transparencia en el reclutamiento y la inclusión de los participantes.

**Figura 1 -:** Diagrama de flujo en el que se ilustra el proceso para seleccionar e incluir participantes

Los resultados del ACL confirmaron que el modelo de tres clases presentó los mejores indicadores de ajuste relativo [AIC=1976,884; aBIC=1982,509; aLMR=89,914; Regresión Lineal Bayesiana (RLB)=92,676]. La entropía correspondiente al modelo con tres clases alcanzó un nivel adecuado (0,82).

En la Tabla 1 se presentan las puntuaciones medias correspondientes al estigma internalizado en cada una de las dimensiones de ISMI-29 y la distribución de las covariables. Entre los pacientes con perfiles latentes de estigma alto hubo un 50% de hombres y el 47,9% se identificó como perteneciente a algún grupo étnico. También presentaron la peor media de ajuste premórbido (13,75), la DUP más prolongada en años (3,85) y los peores síntomas de la muestra (63,45). Por otra parte, el perfil latente de estigma moderado estuvo mayoritariamente conformado por hombres (62,4%) y por personas que se identificaron como pertenecientes a algún grupo étnico (58,4%). También presentaron niveles intermedios de ajuste premórbido (10,82), psicosis no tratada en años (1,88) y síntomas** **(61,83). Finalmente, el perfil latente de estigma bajo estuvo mayormente compuesto por hombres (61,5%) y por personas que se identificaron como pertenecientes a algún grupo étnico (59,6%). Presentó los mejores niveles de ajuste premórbido (9,45), psicosis no tratada en años (1,58) y síntomas (59,54).

**Tabla 1 -:** Estadísticas descriptivas correspondientes a las variables clínicas. Arica, Arica y Parinacota, Chile, 2023

Valor medio (DE*)					
**ISMI** ^†^		**Toda la muestra**	**Estigma alto**	**Estigma moderado**	**Estigma bajo**
Alienación		13,34 (4,22)	2,69 (0,06)	2,218 (0,06)	1,359 (0,06)
Discriminación percibida		11,26 (3,68)	2,87 (0,05)	2,131 (0,07)	1,363 (0,07)
Autoestigma		14,60 (4,71)	2,66 (0,04)	1,974 (0,07)	1,243 (0,06)
Resistencia al estigma		12,80 (2,92)	2,33 (0,06)	2,659 (0,06)	2,867 (0,10)
Aislamiento social		13,45 (5,06)	2,84 (0,07)	2,172 (0,11)	1,270 (0,07)
(DE*)/Frecuencia (%)					
**Covariables**		**Toda la muestra (n=227)**	**Estigma alto (n=98)**	**Estigma moderado (n=77)**	**Estigma bajo (n=52)**
Sexo	Masculino	129 (56,8%)	49 (50%)	48 (62,4%)	32 (61,5%)
	Femenino	98 (43,2%)	49 (50%)	29 (37,6%)	20 (38,5%)
Grupo étnico	Sí	123 (54,2%)	47 (47,9%)	45 (58,4%)	31 (59,6%)
	No	104 (45,8%)	51 (52,1%)	32 (41,6%)	21 (40,4%)
Ajuste premórbido		11,52 (4,45)	13,75 (4,30)	10,82 (4,01)	9,45 (3,70)
Duración de psicosis no tratada		2,46 (6,36)	3,85 (8,76)	1,88 (5,17)	1,58 (3,11)
Síntomas		61,80 (18,91)	63,45 (16,19)	61,83 (21,64)	59,5 (19,43)

*DE = Desviación Estándar; ^†^ISMI = Escala *Internalized Stigma of Mental Illness*

En la Figura 2 se muestra la distribución de los perfiles latentes por medio de un *boxplot* para cada una de las clases y dimensiones de ISMI-29. Se puede afirmar que los sujetos con significativas tendencias autoestigmatizantes y menor resistencia al estigma, más propensos a concordar con estereotipos negativos sobre la esquizofrenia, han sufrido más discriminación y presentan niveles más elevados de aislamiento social (n=98) fueron agrupados en (C1). Por otra parte, las personas con niveles promedio en todas las dimensiones de ISMI-29 fueron incluidas en (C2) (n=77). A su vez, en (C3) encontramos participantes con mayor resistencia al estigma y menores tendencias autoestigmatizantes, capaces de tener una visión positiva sobre sí mismos y la enfermedad, con poca discriminación percibida y mayor participación social (n=52).

**Figura 2 -:** Valores medios correspondientes a los perfiles latentes de estigma internalizado de acuerdo con las dimensiones de ISMI-29

*C3 = Estigma bajo; ^†^C2 = Estigma moderado; ^‡^C1 = Estigma alto

En la Tabla 2 se presentan los resultados de la regresión logística multinomial, examinando la asociación entre las covariables y pertenencia a clases latentes para los perfiles de estigma internalizado. Se utiliza el perfil de estigma bajo como grupo de referencia para la comparación.

**Tabla 2 -:** Resultados de la regresión logística multinomial para las covariables de las clases latentes. Arica, Arica y Parinacota, Chile, 2023

**Grupo de referencia**	**Grupo de comparación**	**Grupo étnico**	**Sexo**	**Duración de psicosis no** **tratada**	**Ajuste premórbido**	**Síntomas**
OR* (IC ^†^ )						
Estigma alto	Estigma moderado	1,09(0,76; 1,56)	0,34(0,04; 2,92)	0,91(0,79; 1,06)	0,87(0,71; 1,06)	0,94(0,88; 1,01)
	Estigma bajo	0,88(0,65; 1,19)	0,33(0,06; 1,71)	0,98(0,89; 1,08)	0,70(0,58; 0,85) ^‡^	0,98(0,93; 1,04)
Estigma bajo	Estigma moderado	1,23(0,86; 1,76)	1,04(0,22; 4,8)	0,93(0,80; 1,08)	1,23(1,00; 1,5) ^‡^	0,95(0,90; 1,01)

Nota: Se utilizó el perfil de estigma bajo como grupo de referencia para la comparación; *OR = *Odds Ratio*; ^†^IC = Intervalo de Confianza; ^‡^Significancia estadística

Tal como se muestra en la Tabla 2, el ajuste premórbido fue el único predictor significativo de pertenencia tanto en el perfil de estigma Alto como en el de estigma bajo. Específicamente, un ajuste premórbido deficiente (OR=0,70 para estigma alto y OR=1,23 para estigma moderado) aumenta la probabilidad de que una persona sea clasificada en los perfiles moderado o alto de estigma internalizado, en comparación con el grupo de estigma bajo.

Esto sugiere que las personas con peor ajuste psicosocial antes de desencadenarse la psicosis son más propensos a sufrir niveles más elevados de estigma internalizado, en comparación con aquellos que presentan mejor ajuste premórbido, que tienen más probabilidades de ser categorizados en el grupo de estigma bajo.

## Discusión

En el Análisis de Clases Latentes (ACL) que se realizó en este estudio se identificaron tres perfiles diferentes de estigma internalizado en personas con diagnóstico de esquizofrenia, a saber: alto, moderado y bajo. La clasificación con tres perfiles demostró ser un modelo efectivo para categorizar a los participantes en función de sus puntuaciones de estigma latente. Contrariamente a nuestra hipótesis, el origen étnico no se erigió como un predictor significativo de pertenencia al perfil de estigma alto. Este hallazgo desafía ciertas presunciones predominantes en la literatura sobre estigma. Si bien en trabajos de investigación anteriores a menudo se enfatizado la relación entre pertenecer a algún grupo étnico y la intensificación del estigma^(1)^ y contrariamente a nuestra hipótesis, padecer esquizofrenia y pertenecer a un segmento social vulnerable como algún grupo étnico^(13)^ probablemente no aumenten los niveles de estigma, rechazando así la combinación de condiciones propuesta como hipótesis. Estos resultados sugieren que la identidad étnica por sí sola no necesariamente intensifica el estigma internalizado en personas que padecen esquizofrenia. Esto implica que, en el contexto de este segmento de la población, padecer esquizofrenia y presentar otros factores como antecedentes psicosociales conjuntamente puede ejercer mayor influencia sobre el estigma internalizado que la identidad étnica de forma aislada.

Un hallazgo clave de este estudio fue el hecho de que el ajuste premórbido se erigió como un predictor significativo tanto para el perfil de estigma alto como para el de estigma moderado. Específicamente, las personas con ajuste premórbido deficiente presentaron más probabilidades de ser categorizados en el grupo de estigma alto, con una asociación ligeramente menor con el grupo de estigma moderado. Estos resultados se condicen con los de estudios anteriores^(14)^, en los que se sugiere que las personas con un funcionamiento psicosocial deficiente antes de desencadenarse la psicosis corren mayor riesgo de internalizar estereotipos negativos. Es plausible que las personas que sufrieron inconvenientes en los dominios social, educativo u ocupacional antes de desencadenarse la psicosis hayan contado con menos recursos para combatir el estigma una vez diagnosticado, lo que los hizo más vulnerables a internalizar prejuicios de la sociedad^(37)^. Es posible que estos individuos también hayan sido más susceptibles a juicios sociales negativos, lo que podría haber contribuido a una mayor internalización de su estigma. Entre las intervenciones para abordar el estigma podemos mencionar las siguientes: Terapia Cognitivo-Conductual, que desafía las creencias negativas y la aceptación; Terapia del Compromiso, que estimula la aceptación del trastorno; y programas de Psicoeducación para generar mayor conciencia y enseñar estrategias de afrontamiento^(38-40)^.

Este hallazgo enfatiza el rol crítico del funcionamiento premórbido en términos de experiencias relacionadas con el estigma y destaca la necesidad de diseñar intervenciones personalizadas dirigidas a mitigar el problema. En las estrategias clínicas se deberían considerar las necesidades únicas de las personas con antecedentes de ajuste psicosocial deficiente antes de desencadenarse la psicosis. Estos pacientes podrían resultar beneficiados al participar en intervenciones diseñadas para mejorar sus mecanismos de funcionamiento y afrontamiento social, lo que a su vez podría reducir la internalización de actitudes negativas con respecto a la sociedad. La significativa asociación entre ajuste premórbido y estigma internalizado sugiere que el desarrollo psicosocial temprano debería ser un punto central a tratar, tanto en trabajos de investigación como en la práctica clínica.

Es más, en el estudio se descubrió que la gravedad de los síntomas, medidos con la Escala PANSS, no fue un predictor significativo de los perfiles de estigma. Esto se condice con otros estudios de investigación en los que se sugiere que la relación entre la gravedad de los síntomas y el autoestigma es compleja y no siempre directa^(36)^. Es posible que diversos factores psicosociales como el ajuste premórbido desempeñen un rol más central en la conformación de experiencias de estigma que la gravedad de los síntomas psicóticos en sí. Este hallazgo exige comprender mejor los factores que ejercen una influencia sobre el estigma, más allá de la gravedad de los síntomas de forma aislada.

Pese a los valiosos detalles que aporta este estudio, es necesario reconocer varias limitaciones. En primer lugar, el diseño transversal restringe nuestra capacidad para evaluar la estabilidad de los perfiles de estigma internalizado a lo largo del tiempo o para determinar si las covariables identificadas (ajuste premórbido, por ejemplo) ejercen algún efecto duradero sobre la evolución del estigma. Realizar estudios de investigación longitudinales permitiría comprender más claramente cómo progresa el estigma internalizado y las distintas influencias ejercidas por diversos predictores sobre la progresión del estigma con el transcurso del tiempo. En particular, un Análisis de Transición Latente (ATL) podría ser un método útil para examinar de qué manera las personas pasan de un perfil de estigma a otro durante diferentes etapas de su tratamiento y recuperación.

Otra limitación es el hecho de que el estudio se enfocó en un entorno clínico específico: centros públicos de salud mental de Chile. Si bien estos centros proporcionan datos importantes sobre el estigma en una población clínica diversa, no es posible generalizar directamente los hallazgos a otros entornos o grupos poblaciones, especialmente los pertenecientes a distintos contextos culturales o sistemas de atención de la salud. En estudios a futuro se deberá investigar el rol del ajuste premórbido y de otros predictores del estigma en distintos entornos geográficos y culturales para validar la solidez de estos hallazgos.

## Conclusión

A modo de conclusión, este estudio destaca la utilidad del Análisis de Clases Latentes al momento de clasificar perfiles de estigma internalizado en personas con esquizofrenia. Los resultados sugieren que, mientras que pertenecer a algún grupo étnico no es un predictor significativo de niveles más elevados de estigma internalizado en este segmento de la población, un ajuste premórbido deficiente sí predice significativamente los perfiles de estigma alto y moderado por igual. Estos hallazgos ponen en relieve la importancia de incorporar los antecedentes psicosociales de los sujetos (su ajuste premórbido particularmente) a intervenciones diseñadas para reducir el estigma. Los individuos con antecedentes de funcionamiento premórbido deficiente podrían requiere intervenciones más personalizadas destinadas a mejorar sus habilidades de funcionamiento y afrontamiento social, lo que podría reducir la internalización del estigma.

En trabajos de investigación a futuro se deberá seguir examinando el rol del ajuste premórbido, la gravedad de los síntomas y otros factores psicosociales en la evolución del estigma. Lo ideal sería recurrir a diseños longitudinales para registrar la naturaleza dinámica de este problema con el transcurso del tiempo. Además, las intervenciones destinadas a reducir el estigma en esquizofrenia deberían personalizarse para abordar estos factores psicosociales y, en futuros estudios, se deberán explorar los métodos más efectivos para tratar el estigma internalizado en diversos subgrupos dentro de este segmento de la población.
